# Surveillance of Arthropod Vector-Borne Infectious Diseases Using Remote Sensing Techniques: A Review

**DOI:** 10.1371/journal.ppat.0030116

**Published:** 2007-10-26

**Authors:** Satya Kalluri, Peter Gilruth, David Rogers, Martha Szczur

**Affiliations:** University of British Columbia, Canada

## Abstract

Epidemiologists are adopting new remote sensing techniques to study a variety of vector-borne diseases. Associations between satellite-derived environmental variables such as temperature, humidity, and land cover type and vector density are used to identify and characterize vector habitats. The convergence of factors such as the availability of multi-temporal satellite data and georeferenced epidemiological data, collaboration between remote sensing scientists and biologists, and the availability of sophisticated, statistical geographic information system and image processing algorithms in a desktop environment creates a fertile research environment. The use of remote sensing techniques to map vector-borne diseases has evolved significantly over the past 25 years. In this paper, we review the status of remote sensing studies of arthropod vector-borne diseases due to mosquitoes, ticks, blackflies, tsetse flies, and sandflies, which are responsible for the majority of vector-borne diseases in the world. Examples of simple image classification techniques that associate land use and land cover types with vector habitats, as well as complex statistical models that link satellite-derived multi-temporal meteorological observations with vector biology and abundance, are discussed here. Future improvements in remote sensing applications in epidemiology are also discussed.

## Introduction

Hematophagous arthropod vectors such as mosquitoes, ticks, and flies are responsible for transmitting bacteria, viruses, and protozoa between vertebrate hosts, causing such deadly diseases as malaria, dengue fever, and trypanosomiasis. Until the early 20th century, vector-borne diseases were responsible for more deaths in humans than all other causes combined. These diseases prevented the development of large areas of the tropics, especially in Africa [1]. Table 1 provides a list of common arthropod vectors, the diseases they carry, and the type of pathogen responsible for the disease. Floods and other natural disasters create environments conducive to the spread of communicable diseases such as malaria, diarrhea, and cholera. Some studies suggest that climate change and increased climate variability are fostering the spread of infectious diseases beyond their traditional geographic domains [2]. For example, West Nile virus, which was previously confined to Africa, Asia, and Europe (i.e., the Old World), has recently spread to North America. The mosquito Aedes albopictus, a vector of both dengue fever and West Nile virus and a native to Asia, has recently established in North America [3]. The “burden” of prominent infectious diseases worldwide transmitted by arthropod vectors is given in Table 2 [4]. Disease burden is expressed in Disability Adjusted Life Years (DALYs), which is the sum of years lost prematurely due to mortality and disability for incidence cases of the disease [5]. One DALY represents the loss of a year of healthy life.

**Table 1:**
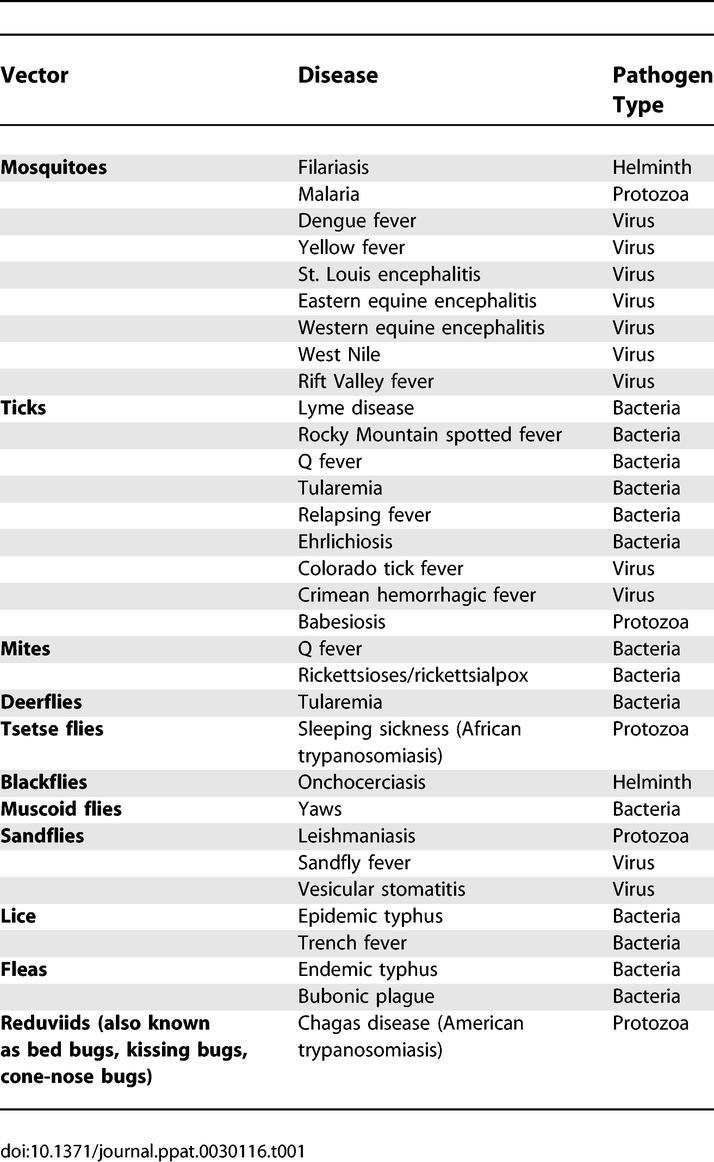
Common Arthropod Vectors, Diseases, and the Type of Pathogen Responsible for the Disease

**Table 2:**
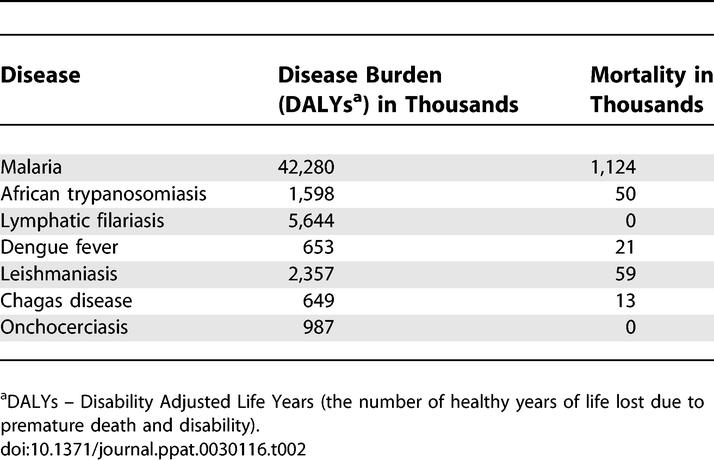
Global Burden of Infectious Diseases Caused Due to Arthropod Vectors [4]

Passive disease surveillance involves voluntary reporting by people who are ill enough to go to a treatment center; such centers are therefore only effective for detection and mitigation after a person has been infected. On the other hand, active disease surveillance, which involves “searching” for evidence of disease proactively through routine and continuous monitoring in endemic areas, could help prevent an outbreak, or slow transmission at an earlier stage of an epidemic. Improved methods are required for forecasting, early detection, and prevention of vector-borne diseases due to the increasing trend of large-scale epidemics such as malaria [6]. Of late, satellite remote sensing technology has shown promising results in assessing the risk of various vector-borne diseases at different spatial scales. Satellite measurements and other remote sensing techniques cannot identify the vectors themselves, but may be used to characterize the environment in which the vectors thrive. Environmental variables such as land and sea surface temperature and amount, type, and health of vegetation can be identified and measured from space. A list of environmental factors that can be mapped through remote sensing and their potential linkages with various diseases has been previously described [7]. Satellites have the ability to detect anomalies and deviations from the normal climate patterns that are conducive to the breeding of disease-carrying vectors such as mosquitoes. Techniques to map disease occurrence and risk from satellite data therefore require at least some understanding of the relationships between a vector-borne disease and the air, land, and water environment in which it occurs. The objectives of this review are to summarize developments in the application of remote sensing techniques for studying infectious diseases in humans due to arthropod vectors and to identify future opportunities for further research.

## Remote Sensing of the Vector Environment

Remote sensing satellites provide continuous measurements of the earth and its environment, and offer a synoptic monitoring capability. Satellite measurements have distinct advantages over ground measurements since they can be collected repeatedly and automatically. In the United States, the National Aeronautics and Space Administration (NASA) is the prime agency responsible for developing new remote sensing technologies and remote sensing satellites, collecting earth system science data to study global change, and developing applications to use remotely sensed data. The National Oceanic and Atmospheric Administration (NOAA) operates a series of weather satellites that collect operational data for weather forecasting and climate prediction. Besides NASA and NOAA, several European Union countries, Japan, Canada, and India have remote sensing satellites that provide global observations. A list of different Earth-observing satellite sensors that are discussed in this paper and their spectral, spatial, and temporal characteristics are given in Table 3.

**Table 3:**
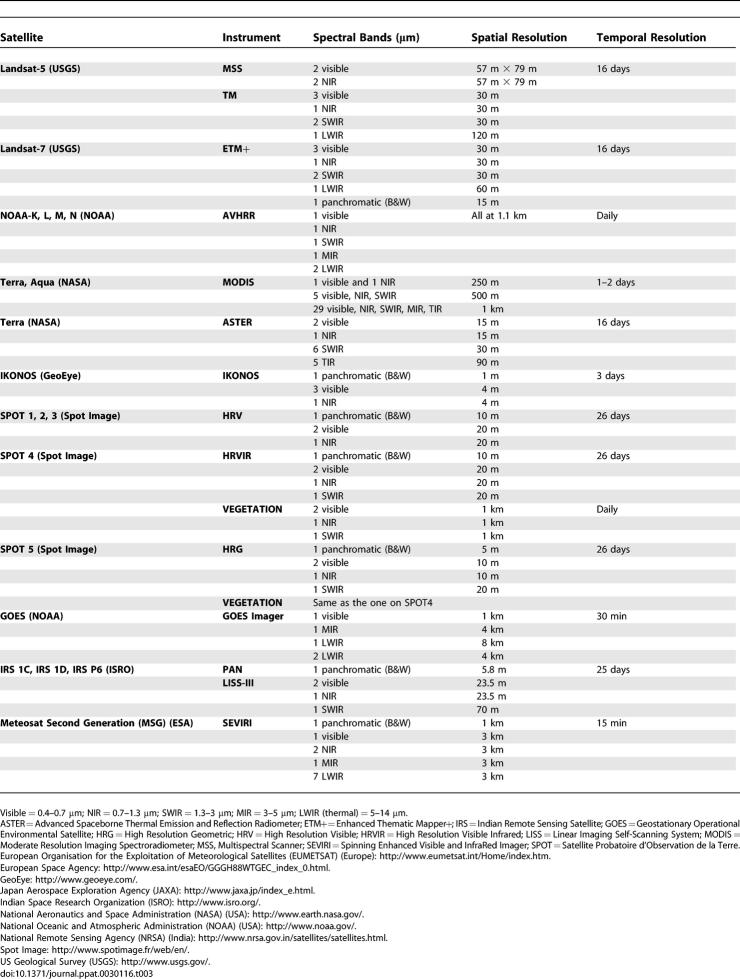
Characteristics of Different Earth-Observing Satellite Instruments Discussed in This Paper That Have Potential Applications in Epidemiology

The use of remote sensing techniques to map vector species distribution and disease risk has evolved considerably during the past two decades. The complexity of techniques range from using simple correlations between spectral signatures from different land use–land cover types and species abundance (e.g., [8,9]) to complex techniques that link satellite-derived seasonal environmental variables to vector biology [10]. While a variety of numerical techniques to create maps of vector distribution in time and space from satellite data are available, only those techniques that aid our understanding of biological processes provide meaningful information in epidemiology and vector control. A review of different modeling approaches for mapping vector and vector-borne diseases is discussed by Rogers [11]. The typical modeling approach is to use either logistic regression or discriminant analysis techniques that investigate associations between multivariate environmental data and patterns of vector presence or absence for mapping vectors and vector-borne diseases. Both of these methods are capable of predicting the a posteriori probability of the presence of the dependent variable (e.g., either the vector or the disease) from a set of independent variables (e.g., climate and land cover data) and can be used to make risk maps from sample data sets (i.e., training datasets) based on the observed similarity of environmental conditions to sites. The choice of techniques should be able to accommodate both categorical (e.g., disease presence or absence) as well as continuous data (e.g., surface temperature data) spatial data.

The approach in developing remote sensing applications in epidemiology depends on the spectral, spatial, and temporal characteristics of remote sensing measurements. Environmental satellites that collect earth observations daily or several times a day of the same geographic region (e.g., NOAA's Polar Operational Environmental Satellites and Geostationary Operational Environmental Satellites) are ideally suited for collecting rapidly changing meteorological variables such as atmospheric moisture and surface temperature. Data from these satellites are valuable in modeling the climate-dependent vector life cycles and sustainability. In general, operational environmental satellites collect more frequent observations at a coarser spatial resolution (∼1 km), over large geographic regions, and are cheaper to acquire compared to images from other land imaging satellites such as Landsat or IKONOS that have less frequent revisit capability but have a higher spatial resolution (30 m or higher). A summary of the availability of environmental satellite data for mapping infectious diseases can be found in [12]. A combination of high spatial resolution data for land use and land cover classification and frequent coarse resolution environmental satellite data for monitoring environmental variability would be ideal for studying surface climate conditions for modeling vector populations.

Application of remote sensing data in epidemiology involves retrieving environmental variables that characterize the vector ecosystem such as land cover, temperature, humidity or vapor pressure, and precipitation. Measurements of Earth's surface reflectances and temperature can be made directly from satellites. However, measuring meteorological and climate variables near the surface is more difficult, and frequently, empirical methods are used. Because of complexities and limitations in estimating meteorological variables through remote sensing, proxy variables such as vegetation indices that measure the abundance, spatial extent, and dynamics of vegetation are used as a surrogate indicator of climate variability in epidemiological studies (e.g., [13]). The Normalized Difference Vegetation Index (NDVI), which exploits the strong contrast in the reflectance of vegetation in the red and near infrared wavelengths, is a commonly used index to study vegetation dynamics (e.g., [14]). Since vegetation dynamics are influenced by variations in climate, strong correlations between vegetation indices and climate variables can be found. A combination of vegetation indices, surface reflectance, and temperature measurements have been used by epidemiologists to model vector ecosystems (e.g., [10]). An earlier discussion of remote sensing techniques used in the study and control of invertebrate hosts and vectors for diseases can be found in [15].

## Remote Sensing Studies in Arthropod Vector-Borne Diseases

### 

#### Mosquitoes.

**Figure 1:**
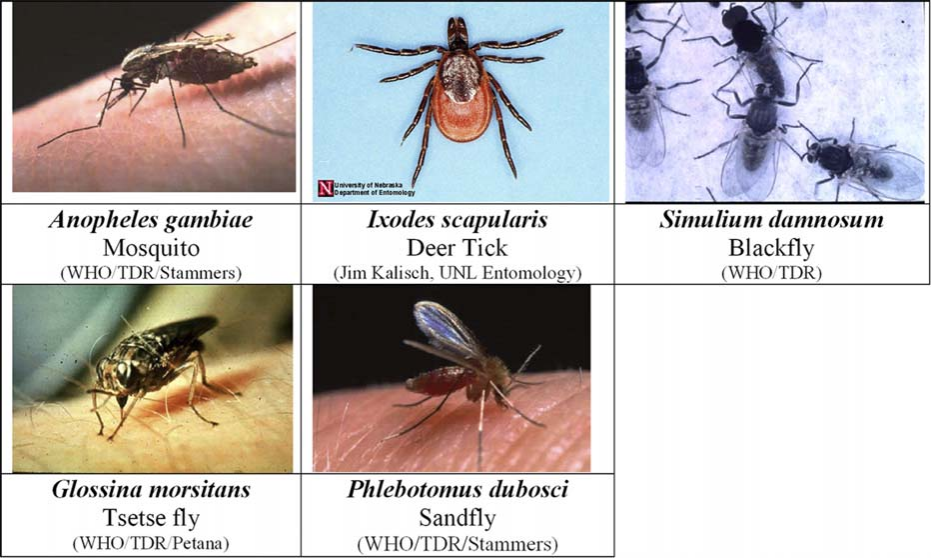
Arthropod Vectors That Are Discussed in This Paper

*Diseases and vector habitats.* Mosquitoes (Figure 1) are found throughout the world, and mosquito-borne diseases are among the world's leading causes of illness and death. Despite great strides over the last 50 years, the World Health Organization estimates that more than 300 million clinical cases of mosquito-borne illnesses occur each year. There is a long history of developing disease transmission models by mosquitoes (e.g., [16,17]) and a good summary is provided by Anderson and May [18]. Several factors, such as seasonality, proximity to breeding grounds, vector density, biting rates, and proportion of infectious mosquitoes, contribute to the spread of mosquito-borne diseases.

Mosquitoes require still or slow moving water for completing the larval and pupal stages of their life cycle. Both natural as well as man-made environments of stagnant water are conducive to their breeding. For example, in Asia and America, Aedes aegypti breeds primarily in man-made water containers such as automobile tyres, metal drums, recycling containers, and domestic water storage containers, whereas in Africa, they breed in tree holes and leaf axils [19,20]. Temperature, precipitation, and relative humidity are the three main factors that determine the abundance of mosquitoes and the prevalence of mosquito-borne diseases such as malaria [21]. The optimum temperature for mosquito development of tropical species is 25–27 °C [22], and there is a strong temperature dependence of the development of the parasites within the mosquito vectors. For example, the time needed for the sporozoites of Plasmodium falciparum to reach the salivary glands of mosquitoes is inversely proportional to the air temperature with a difference of 14 days between 30 °C and 10 °C [23]. Mosquito-borne diseases such as malaria are seasonal and case numbers correlate well with rainfall patterns [23]. However, too little rainfall creates fewer breeding habitats, and too much rainfall tends to wash away the mosquito eggs [24]. Irrigated agricultural areas such as paddy fields in Asia and inland tidal waters are also very favorable for the breeding of some mosquito species.

*Remote sensing studies of mosquito-borne diseases.* Mosquito-borne diseases are prevalent throughout the world, and remote sensing applications in epidemiology have been most widely used to study mosquito-borne diseases. A good summary of mosquito biology and methods to map their habitats from satellites can be found in [25].

Initial studies largely focused on identifying mosquito breeding habitats such as marshes and wetlands through land-use and land-cover mapping using remote sensing data (e.g., [26–31]). For example, Landsat data was used to determine green leaf area index (LAI) over 104 rice fields, and these measurements were compared to larval counts of Aedes freeborni at the edge of the fields and the minimum distance from the center of each field to the nearest livestock pastures that provide the blood-meal source [8,32]. This analysis showed that fields that are near pastures that have high LAI and tiller density produce large numbers of mosquitoes, and fields with low LAI that are further from the pastures have lower numbers of mosquitoes. A combination of spectral measurements from the satellite data and distance measurements to pastures were used in discriminant analysis to identify high mosquito producing areas with 90% accuracy.

Multispectral data from the SPOT (Satellite Probatoire d'Observation de la Terre) satellite was used to map the probability of mosquito presence in Belize by Roberts et al. [33]. This study measured the distance of houses from waterways, altitude above specified waterways, and amount of forest between houses and waterways. Each site was then ranked as high, medium, or low for probability of mosquito infestation based on thresholds of distance, elevation, and forest cover. Areas that were closest to the water in both distance and altitude with no intervening forests were assigned the highest probability of mosquito presence around humans. Their results showed that Anopheles pseudopunctipennis was present in 50% of all the high probability locations and absent in all the low probability locations.

Anopheles darlingi larval habitats were surveyed in Belize by sampling along the Sibun River in Belize and compared with land cover classification from SPOT and IKONOS data [34]. Ground survey showed a strong correlation between positive larval habitats and debris from trees such as fallen trunks, branches, and root systems compared to other landscape features. However, no such correlations could be made between positive larval habitats and different land cover types such as forests and orchards or landscape features such as river bends where one would expect to find fallen tree detritus. Overhanging spiny bamboo trees were one of the major sources of floating detritus mats for larval breeding, but the satellite imagery did poorly in discriminating bamboos from other vegetation during classification in this study. The only association between positive and negative A. darlingi sampling sites was their distance to the nearest houses, where the negative sites were 162 m further away from homes compared to the positive sites. The authors conclude that the high cost of high spatial resolution imagery to identify houses along a river would make this technique unsuitable for developing countries. Nevertheless, the use of high spatial resolution data for mapping land use, land cover, and hydrological features to identify suitable vector habitats continues to be a popular approach (e.g., [35]).

Advances in understanding of correlations between multispectral satellite measurements and meteorological variables such as rainfall and temperature have led to the development of statistical models of mapping vector habitats using relationships between satellite-derived climatology and vector ecology. Rift Valley fever (RVF) is a mosquito-borne disease that affects humans and animals in various parts of Africa. Mosquito breeding habitats in East Africa, known locally as “dambos”, are frequently flooded during periods of heavy rainfall, which leads to a build-up of mosquito populations and subsequent RVF outbreaks. Since NDVI is known to be correlated with variations in rainfall, variability of NDVI has been shown to be associated with RVF incidence [36]. A discussion on the use of temporal profiles of NDVI and cold cloud duration (CCD) measurements from satellites in models of mosquito distribution and malaria in Africa is found in [37]. Sea surface temperature (SST) variations indicate that El Niño Southern Oscillation and concurrent SST elevations in the Pacific and Indian oceans are correlated with increased rainfall in Eastern Africa. When SST data and NDVI were used together as the predictor variables, the incidence of RVF could be forecast up to 5 months in advance [38,39].

Temporal Fourier processed measurements of NDVI, land surface temperature, and CCD were used by Rogers et al. [10] to capture the seasonal climatology of the African landscape. Fourier processing of these variables reduces the dimensionality of the data set while retaining crucial information about habitat seasonality (information that is lost by other methods of data reduction, such as Principal Components Analysis). The Fourier processed images were used in discriminant analytical models to describe both the distribution of five important species in the Anopheles gambiae complex of species in Africa and the risk of malaria as captured by the entomological inoculation rate (EIR, the number of infectious bites per person per year). Relationships observed between EIR and satellite data were used to make a predictive map of EIR across the continent. Similar techniques were used to create risk maps of West Nile virus in the United States [40] and maps of global distributions of yellow fever and dengue [41]. The predicted risk maps showing the maximum likelihood, posterior probability of disease presence or absence had an average kappa index between model predictions and observations of 0.742 for yellow fever and 0.700 for dengue.

**Figure 2:**
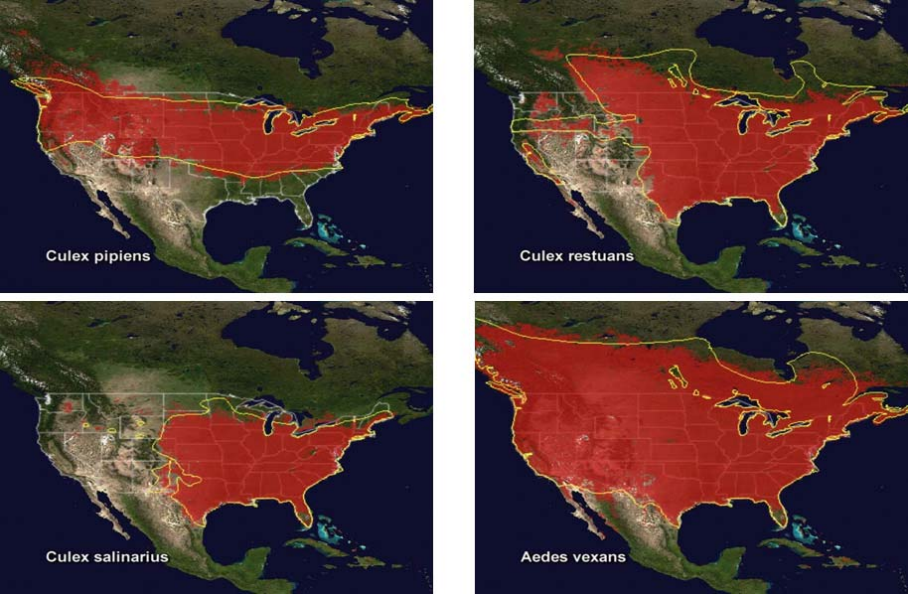
Maps Showing the Potential Distribution of Four Species of Mosquitoes in the United States Distribution predicted using satellite derived environmental data is in red, and recorded distribution is outlined in yellow. Image courtesy: TALA Research Group–University of Oxford.

Figure 2 shows the potential distribution of four species of West Nile virus–carrying mosquitoes in the United States derived using the techniques described by Rogers et al. [40]. Note the close agreement between the geographic extent of distribution derived from satellite data and the recorded distribution on the ground.

#### Ticks.

*Diseases and vector habitats.* Ticks are responsible for transmitting a variety of pathogens, including protozoa, rickettsia, bacteria, and viruses, to both humans and livestock [42], which causes substantial economic damage worldwide [43,44]. Lyme disease, Rocky Mountain spotted fever, ehrlichiosis, and babesiosis are some of the more common diseases in humans that are transmitted by ticks. Lyme disease is the most common vector-borne disease of humans in the United States and Europe [45,46].

Unlike other arthropod vectors such as mosquitoes, ticks are hematophagous only once per life cycle stage, taking large blood meals equivalent to 10–100 times their body weight [47]. The primary hosts of Ixodes scapularis (Figure 1), the black-legged tick that carries the bacteria Borrelia burgdorferi, which causes Lyme disease in humans, are the white-footed mice (Peromyscus leucopus) and white-tailed deer (Odocoileus virginianus) [45]. During their life cycle, different Ixodid tick species may use one, two, or three different host species [48]. In their free-living stages, most tick species have specific requirements in terms of microclimate and tend therefore to be picked up only by those hosts that frequent the habitats providing such conditions [48–51]. Tick populations are abundant in deciduous forests with leaf litter and ecosystems with shrubs and tall grasses in temperate climates with high relative humidity. Lyme disease shows strong seasonality, with peaks occurring during the summer and fall months when the nymphs are most active [52]. However, changes in the incidence of tick-borne diseases cannot always be related to climate change; changes in other elements of complex epidemiological cycles may also play a part. For example, Lyme disease within the United States is concentrated mostly in the northeastern Atlantic states [53], and the spread of this disease seems to be closely related to an increase in the population of the white-tailed deer [54], an increase due to a diminution of hunting pressure and an increase in woodland in close proximity to human habitation.

*Remote sensing studies of tick-borne diseases.* A land cover map derived from a Landsat Thematic Mapper (TM) image of Guadeloupe, French Windward Islands, was used by Hugh-Jones [55,56] to discriminate four grazing regions with different levels of cattle tick Amblyomma variegatum infestation in 103 cattle herds. The four classes were lightly infested dry meadows, moderately infested foothills, heavily infested dry scrub, and rocky grasslands. The spatial variability of pixel values within individual grazing fields also correlated well with tick density; areas where the landscape was variable had more ticks compared to homogenous areas.

Ticks exhibit strong seasonal dependence of mortality and disease transmission, which can be related to temperature and vegetation conditions [47]. A critical factor for egg laying and larval development is temperature and humidity. Since these factors can be related to NDVI, multitemporal NDVI measurements from the Advanced Very High Resolution Radiometer (AVHRR) have been correlated with mean mortality during the life cycle between the adult female and subsequent larval stages in Burundi, Uganda, and Tanzania [57].

Using environmental variables obtained at the residences of Lyme disease patients in Baltimore County, Maryland, from 1989 through 1990 within a geographic information system (GIS), Glass et al. [58] showed that many suitable tick habitats in the northeastern United States coincide with residential properties close to wooded areas. Brightness, greenness, and wetness determined from Landsat data [59] were used along with elevation and land cover classes to determine risk for Lyme disease on 337 residential properties in two communities of suburban Westchester County, New York [60]. Spatial analysis of Lyme disease risk indicated that high-risk properties had a greater abundance of green vegetation and/or a higher proportion of woods than lower and no-risk properties, which is consistent with field-based studies of the landscape ecology of Lyme disease.

Ticks are not highly mobile in nature, and they depend on their hosts (e.g., the white-footed mouse) for movement over large distances. However, not all environments that a host might inhabit are suitable for ticks to survive and reproduce. Therefore, the presence of an adequate host population by itself is not a risk factor for the transmission of Lyme disease. To determine what specific environments are conducive for the survival of I. scapularis in the upper Midwest, a survey was conducted of tick populations by collecting samples from vertebrate hosts on the ground, and environmental data from each collection site was compared with tick abundance [61]. Land cover data derived from Landsat TM data was used with maps of soils, geology, elevation, and climate to determine significant associations between tick presence and environmental variables. Discriminant analysis was used to determine the significant environmental factors that differentiate positive and negative tick sites. Logistic regression analysis was used to create maps of habitat suitability for I. scapularis. Areas that had high suitability coincided with the incidence of Lyme disease in Wisconsin. The results of the logistic regression (83.9% classification accuracy) were in agreement with the discriminant analysis (85.7% classification accuracy) and both techniques showed that soil order and land cover were the dominant factors for tick presence.

Using NDVI and surface temperature measurement from AVHRR between 1982 and 2000, Estrada-Pena [62] showed that favorable tick habitats within the United States are increasing. A reduction in biodiversity due to deforestation and forest fragmentation could lead to an increase in the density of the white-footed mouse, and ticks feeding on the white-footed mouse have a higher probability of being infected with B. burgdorferi than any other host [63].

Environmental data derived from satellites was used by Randolph and Rogers [64] to differentiate the eco-climatic zones of six tick-borne flaviviruses. Since only certain environments that can sustain the appropriate natural life cycles of the vector can support the virus transmission cycles, this study suggests that climate may have played a role in directing the evolution of the flaviviruses. By understanding the biotic and abiotic constraints imposed on the evolution and mutation of vector-borne microbes, it may be possible to predict the non-evolutionary emergence of vector-borne diseases under dynamic eco-climatic conditions with a detailed knowledge of vector biology and transmission conditions.

#### Black flies.

*Diseases and vector habitats.* Blackflies are about 1–6 mm in length and are amongst the smallest blood-sucking Dipterans. They usually breed in well-aerated water bodies such as swiftly moving shallow mountain torrents [48], or sunlit, fast flowing rivers in the tropics [65]. Onchocerciasis or river blindness is caused by the helminth Onchocerca volvulus of the family Filariidae, whose larvae are transmitted between humans by blackflies of the family Simuliidae (Figure 1). Onchocerciasis is the second leading cause of blindness in the world with 96% of the affected people living in 30 countries in sub-Saharan Africa (and Yemen), with the remainder in six countries in Latin America [66]. Several programs such as the Onchocerciasis Control Programme in West Africa [67], the Onchocerciasis Elimination Program for the Americas [68], and the African Programme for Onchocerciasis Control [69] target this dreadful disease which, because its impact is greatest in mature individuals (i.e., the agriculturally productive sector of societies), has a disproportionate effect on human communities.

*Remote sensing studies of diseases due to black flies.* The African Programme for Onchocerciasis Control developed a program called Rapid Epidemiological Mapping of Onchocerciasis (REMO) to enable communities at high risk to be quickly and cheaply identified and mapped for priority treatment. However, when ivermectin, the drug used to treat onchocerciasis is administered to patients who are also infected with Loa loa, fatal complications have been reported [70]. Therefore, before Ivermectin could be distributed, WHO wanted to map areas with L. loa infection so that drug administration protocols could be modified to avoid complications. Flies of the genus *Chrysops* (family Tabanidae) that carry the cutaneous filarial parasite L. loa, causing Calabar swellings in humans, are associated with forest and forest fringe habitats with larval stages restricted to wet, organically rich and muddy low-lying areas within the forests [71]. Using forest cover and landcover classes derived from AVHRR along with soil and topography data, the prevalence of L. loa in six African countries was predicted, and the results were compared with priority areas defined by the REMO project [71]. About 50% of the variation in prevalence rates of the infection could be explained by the satellite-derived environmental factors. There were extensive areas of agreement between the satellite derived maps and areas that were identified by REMO as high priority treatment areas in Cameroon and the Democratic Republic of Congo.

#### Tsetse flies.

*Diseases and vector habitats.* Tsetse flies (genus *Glossina*), which are found only in Africa, transmit various protozoan parasites of the genus *Trypanosoma*, which cause sleeping sickness in humans and “nagana” in domestic animals. There are 30 species or subspecies of tsetse, classified into three groups: the *fusca* group, found mostly in forests, the *palpalis* group, found in forests and riverine vegetation, and the *morsitans* group, found in woodland areas of savannah regions. Human sleeping sickness is caused by *Trypanosoma brucei gambiense* and *Trypanosoma brucei rhodesiense*, which threaten up to 60 million people in 36 countries of sub-Saharan Africa [72]. *T. b. gambiense* is usually transmitted by tsetse of the *palpalis* group and occurs mostly in cultivated lands within proximity of pools of water in western and central Africa; it is a disease adapted to humans, although other animals—especially domestic ones—may be important reservoir hosts. *T. b. rhodesiense*, which is the more virulent of the two, is usually transmitted by tsetse of the *morsitans* group and occurs primarily in the savannah woodlands of eastern and central/southern Africa [13,73]; it is a disease that occurs naturally in a large number of domestic and wildlife hosts [74]. In humans, as the disease progresses, the parasite crosses the blood–brain barrier and invades the central nervous system, causing neurological problems [75]. Compared to other arthropod vectors such as mosquitoes, tsetse flies have a very low reproductive rate and a longer life expectancy. The mortality and reproductive rates of tsetse flies are highly dependent upon microclimatic conditions, with high survival rates in cool, moist areas [76]. Animal trypanosomiasis occurs more or less throughout the area of Africa inhabited by one or more species of tsetse (an area of approximately 10 million km^2^), while human sleeping sickness, which has a much higher threshold for transmission [77], occurs only in a relatively few, but very persistent, disease foci.

*Remote sensing studies of diseases due to tsetse flies*. Predictive, process-based models of disease transmission based on the biology of the insect vectors are in general more robust than statistical models, which do not necessarily describe causal relationships between the predictor variables and the predicted phenomena. Air temperature and vapor pressure deficit from climatology records have been successfully used to model the birth and death rates, and density, of tsetse flies over Africa [76]. Since these environmental variables can be related to NDVI, land surface temperature, and cold cloud top temperature duration (CCD) observed from meteorological satellites, techniques have been developed for modeling tsetse distribution in Africa using multi-temporal satellite data [78–80]. The techniques used in these analyses include Fourier analysis of the time series data to reduce the dimensionality while capturing the seasonality of variables, and classifying the images using linear and non-linear discriminant analysis to predict species distribution. These methods were able to predict the distribution of tsetse with accuracies of greater than 80%. Among the different variables used in modeling tsetse distribution, NDVI was considered to be the most important variable, followed by CCD, surface temperature, and elevation. Analysis of habitats and species' distributions from these studies indicated that different species of tsetse flies are differently but closely adapted to local climate conditions. In some cases, the presence or absence of flies was shown to be dependent upon temperature differences of 1 °C or less, and the importance and number of environmental variables in determining tsetse distribution differed from region to region. A review of different methods used in tsetse modeling from satellite data is provided by Rogers [80], and this review contains the first ever satellite-driven, process-based model of disease transmission (trypanosomiasis) based upon a satellite-driven, processed-based model for its insect vector (tsetse flies).

#### Sandflies.

*Diseases and vector habitats*. Visceral leishmaniasis (kala-azar), mucocutaneous leishmaniasis, and cutaneous leishmaniasis are three diseases caused by *Leishmania* protozoa (close relatives of trypanosomes) that are spread through the bite of about 30 different species of sandflies of the subfamily Phlebotominae (super-family Psychodoidea) [81]. Sandflies feed mostly at night, when they are most active, and breed in dark, humid environments with organic matter that serves as food for the larvae [48]. Studying their life cycle is difficult because the larvae, which are tiny, are very hard to find, even in areas of high disease prevalence [82]. Sandflies are restricted to tropical and temperate climates (hot and humid), and therefore leishmaniasis is endemic to these areas, which include northern Africa, the Middle East, parts of Europe, and central South America [73]. Leishmaniasis is primarily a zoonotic disease, affecting mostly rodents and dogs, and humans are incidental hosts. Factors such as deforestation, population migration from endemic rural areas, and increased population in areas with low sanitation have caused a resurgence of leishmaniasis by increasing the contact between the vectors and the hosts [83].

*Remote sensing studies of diseases due to sandflies.* Leishmaniasis transmitted by sandflies was reported as a health hazard for troops deployed in the Middle East both during World War II and in the 1991 Persian Gulf War. The spatial distribution of sandflies is, however, not well understood, mainly because the larvae are so difficult to find. The distribution of Phlebotomus papatasi using NDVI and meteorological data in the Middle East has been modeled [84]. In this study [84], published reports of leishmaniasis and sandfly fever in the Middle East were used to determine the presence of sandflies and their location. Meteorological data were collected on the ground from 114 weather stations in nine countries, including Saudi Arabia, Kuwait, Iran, and Iraq. Meteorological data were not available for all of the locations that had leishmaniasis and sandfly fever. The objective, therefore, was to develop a technique that would determine the probability of disease for those areas where there were no weather data. Using discriminant analysis, the probability of vector occurrence was determined for all 114 locations that had weather data. For those areas that were determined to be positive for sandfly presence, NDVI measurements from AVHRR were analyzed for a 12-year period from 1982. Analysis indicated that the range of NDVI that was associated with vector presence was 0.0–0.06, and this information was used to create a map showing the areas where sandflies could be present. This map was able to identify accurately all the areas where sandflies were present, including those areas that had no meteorological data.

Comparison of sandfly (P. argentipes) densities with different land use land cover types derived from IRS LISS3 (Indian Remote Sensing Satellite Linear Imaging Self-Scanning System 3) data over two are areas that were endemic and non-endemic to visceral leishmaniasis (kala-azar) in Northern India showed that the endemic areas had a higher percentage of water bodies [85]. The endemic areas also had a higher proportion of marshes compared to the non-endemic areas, and there were also significant differences in vegetation and soil types among the two areas. Succulent vegetation was more prominent in endemic areas, whereas non-endemic areas had predominantly thorny and hard-stemmed plants. In endemic areas, the vector density (as described by the man hour density) was higher during the summer (March–June) and rainy seasons (July–October) compared to non-endemic areas. This study was useful for the health authorities in prioritizing their visits to specific sites.

## Future Prospects

Improvements in both sensor capabilities and data processing algorithms are enabling estimates of parameters such as precipitation from space directly rather than using surrogate variables. The availability of moderate resolution imagery from new NASA satellites such as Terra and Aqua at up to 250-m spatial resolution offers improved capabilities in modeling epidemiology from space at a finer spatial resolution than AVHRR. Technologies such as global positioning systems and GIS provide geolocation and mapping capabilities on the ground at unprecedented accuracies, making it possible to merge more accurately ground observations of vector demographics and disease incidence with satellite data. A wide choice of powerful image processing, GIS and statistical software packages are available within an affordable desktop computing environment, making it feasible for epidemiologists and biologists to experiment with new spatial analysis techniques. Anthropogenic factors such as land cover conversion and increase in greenhouse gases are resulting in global climate change, which could expand the ecosystem boundaries of disease-carrying vectors, resulting in an increase in infectious diseases [86]. Maps showing seasonal risks of vector-borne diseases will be necessary to monitor the impacts of global change on vector ecologies. Our ability to forecast climate trends and events such as El Niño and La Niña and their impact on rainfall patterns in regional environments, combined with an understanding of vector-environmental relationships, will help us to improve forecasts of epidemics. Automation of satellite data processing could lead to the generation of risk maps in near real-time to warn local health care professionals.

A complex set of biotic and abiotic factors influence the emergence and spread of vector-borne diseases. While it is not possible to predict the evolution of new vector-borne pathogens, remote sensing techniques can aid in determining the influence of abiotic environmental factors on their spread. Although multitemporal satellite data are available for an extended time period, the availability of georeferenced and spatially explicit disease data for the same temporal record is still less common, especially in developing countries that have a high burden of vector-borne diseases. Other issues that are impacting the routine use of remote sensing in epidemiology include accessibility to high resolution and low-cost imagery [87,88], as well as issues with data continuity in terms of consistencies in spatial, spectral, and temporal resolutions among satellite sensors during different years. Unforeseen problems, such as the failure of Landsat 7 satellite and delays in the launch of new earth observing satellites such as the National Polar-orbiting Operational Environmental Satellite System, as well as shifting priorities of space agencies, could impact our ability to routinely use satellite data in epidemiology and other applications [89]. However, these issues with remote sensing data are not unique to epidemiological applications alone. It should be noted that the use of remote sensing techniques for modeling and forecasting vector-borne diseases is an emerging field, and a sustained use of these applications could be ensured by collaboration between remote sensing scientists and epidemiologists from the onset of research projects [87].

Remotely sensed environmental variables such as air temperature, humidity, and rainfall should be processed and made available to epidemiologists in real-time and in a format that they can readily use as inputs to their modeling by agencies and organizations that collect and archive satellite data. Within the United States, remote sensing data have potential applications in modeling risk from West Nile fever, dengue fever, and Lyme disease. The potential for epidemic dengue transmission within the United States still exists because of the presence of A. albopictus and *A. aegypti* mosquitoes that transmit the disease [1]. Application of remote sensing techniques to map areas at risk for dengue fever within the United States is yet to be done. The predictive maps of diseases need to be verified on the ground for accuracy. While several studies have shown correlations between global climate change and variations in the number of people infected with a particular vector-borne disease, it not yet clear how much change in disease would have occurred without environmental change. Efforts therefore should also focus on the development of stochastic, process-based models that rely on vector biology as predictors of diseases and their risk, instead of statistical models that do not clearly explain causal relationships between satellite data and disease. Nevertheless, simple statistical models could be a good starting point for linking the limited number of environmental variables that can be derived from satellite data with spatial and temporal patterns of diseases and vectors. Simple statistical models could help deduce the epidemiological processes from an analysis of the observed spatial patterns of the disease and the environment.

Remote sensing of vector ecosystems and interpretation of these patterns is likely to provide both challenges and opportunities in epidemiology. In addition to vector biology, social and behavioral patterns such as the time spent outside, which increases risk of exposure to anthropophilic vectors, types of house constructions, the use of nets and other repellents, as well as the availability of basic sanitation and primary healthcare facilities, which are related to socioeconomic conditions, are important in disease prevention and control. Disease patterns have been shown to be closely linked to poverty and social inequalities [90]. These factors cannot be inferred from remote sensing techniques alone.

While the studies reviewed in this paper demonstrate the efficacy of remote sensing and other geospatial technologies in disease surveillance, there are however, several factors that need to be considered for these technologies to be routinely adopted for public health management. These include the availability of resources for gathering, processing, and modeling geospatial data, training of personnel on the proper interpretation of results, cost effectiveness of these surveillance techniques, and the continuous availability of remote sensing data in a timely manner. It should also be emphasized that the allocation of resources for these novel monitoring techniques should not come at the cost of basic disease prevention and management activities at the community level.

## Abbreviations

AVHRR: Advanced Very High Resolution Radiometer
CCD: cold cloud duration
DALY: Disability Adjusted Life Year
EIR: entomological inoculation rate
GIS: geographic information system
LAI: green leaf area index
NASA: National Aeronautics and Space Administration
NOAA: National Oceanic and Atmospheric Administration
NDVI: Normalized Difference Vegetation Index
RVF: Rift Valley fever
SPOT: Satellite Probatoire d'Observation de la Terre
SST: sea surface temperature
TM: thematic mapper
